# The Causal Relationship between Angina Pectoris and Gout Based on Two Sample Mendelian Randomization

**DOI:** 10.1155/2024/4564596

**Published:** 2024-04-09

**Authors:** Jian Xiong, Yuxin Sun, Hui Huang, Yu Liu, Fayang Ling, Yin Wei, Qianhua Zheng, Wenchuan Qi, Fanrong Liang

**Affiliations:** ^1^Chengdu University of Traditional Chinese Medicine, Chengdu, Sichuan 610075, China; ^2^Guangxi University of Traditional Chinese Medicine, Nanning, Guangxi 530001, China

## Abstract

**Purpose:**

Two-sample Mendelian randomization (MR) was conducted to assess the causal relationship between angina pectoris and gout. *Material and Methods*. Based on genome-wide association studies, single nucleotide polymorphisms (SNPs) that were closely associated with gout were selected from the UK Biobank–Neale Lab (ukb-a-107) as genetic instrumental variables. Considering that gout is characterized by elevated blood uric acid levels, SNPs related to blood uric acid levels were screened from BioBank Japan (bbj-a-57) as auxiliary gene instrumental variables. SNPs closely associated with angina pectoris onset were screened from the FINN dataset (finn-b-I9_ANGINA) as outcome variables. Two-sample MR was conducted, with inverse variance weighting (IVW) of the random effects model as the primary result, along with the weighted median method (WME) and the MR-Egger regression method. To further confirm the causal relationship between angina and gout incidence, a meta-analysis was conducted on the IVW results of the ukb-a-107 and bbj-a-57.

**Results:**

The odds ratios and 95% confidence intervals of the IVW, WME, and MR-Egger results of ukb-a-107 were (OR = 33.72; 95% CI: 2.07∼550.38), (OR = 57.94; 95% CI: 2.75∼1219.82), and (OR = 96.38; 95% CI: 0.6∼15556.93), respectively. The *P* values of IVW and WME were 0.014 and 0.014 (both <0.05), respectively, indicating that the development of angina pectoris was significantly associated with the incidence of gout. The odds ratios and 95% confidence intervals of the IVW, WME, and MR-Egger about bbj-a-57 were (OR = 1.20; 95% CI: 1.07∼1.34), (OR = 1.19; 95% CI: 1.02∼1.38), and (OR = 1.30; 95% CI; 1.06∼1.60), respectively. The *P* values of IVW, WME and MR-Egger were 0.001, 0.027 and 0.017 (all <0.05), respectively, indicating a significant correlation between angina and blood uric acid levels. Scatter plots of ukb-a-107 and bbj-a-57 showed that the causal association estimates of the IVW, MR-Egger, and weighted median methods were similar and that the MR results were accurate. Funnel plots and the MR-Egger intercept of ukb-a-107 and bbj-a-57 showed the absence of horizontal pleiotropy. The leave-out sensitivity analysis results of ukb-a-107 and bbj-a-57 are stable. The meta-analysis of IVW results for ukb-a-107 and bbj-a-57 showed (OR = 1.20; 95% CI: 1.07–1.34, *P*=0.02), confirming that gout characterized by high blood uric acid levels significantly increases the risk of angina attacks.

**Conclusions:**

This MR study found a clear causal relationship between angina pectoris and gout, which increases the risk of angina pectoris.

## 1. Introduction

Gout is a metabolic disease that results from the deposition of urate crystals in joints and cartilage tissues, thereby triggering inflammation and pain, and is associated with purine metabolism disorders and/or uric acid excretion reduction, which results in excessively high blood uric acid concentrations (>0.48 mmol/L) [1]. The clinical manifestations of gout include acute exacerbations of arthritis, gouty tophus formation, and chronic gouty arthritis with tophi, and severe cases can lead to joint disability and renal insufficiency. Gout is often accompanied by other metabolic syndrome-related manifestations, such as cardiovascular disease [2–4], dyslipidaemia, and type 2 diabetes mellitus. According to pathological anatomy and the observed pathophysiological changes, angina pectoris has a clinical phenotype of coronary heart disease, which is related to the atherosclerosis of coronary arteries causing luminal narrowing or occlusion. Moreover, an increase in myocardial load results in temporary ischaemia and myocardial hypoxia, which leads to the patient developing clinical manifestations such as chest tightness, chest pain, and shortness of breath [5, 6].

The incidence of gout and angina pectoris is currently rising year over year [7, 8]. The prevalence of gout has increased worldwide in recent decades, with the global statistical prevalence of gout ranging from 0.1% to 10.0% [9]. Over the past decade, deaths due to cardiovascular disease have increased globally by 12.5%, accounting for approximately one-third of the overall deaths worldwide [10]. Gout is strongly associated with blood uric acid, and elevated blood uric acid levels form an important pathological basis for gout pathogenesis. The association between serum uric acid levels and cardiovascular risk has been studied for decades, and some clinical studies have shown that serum uric acid is a predictor of cardiovascular disease prognosis and that elevated serum uric acid levels may be associated with increased risks of cardiovascular disease and death [11]. A case-control study published in JAMA by the Edoardo Cipolletta team at the University of Nottingham showed that gout is an independent risk factor for cardiovascular disease^【2】^–the incidence of gout was found to be associated with an increased incidence of short-term cardiovascular events, and patients who experienced cardiovascular events had a significantly increased incidence of gout during the first 60 days and the first 61–120 days compared with those who did not experience cardiovascular events (0–60 days, OR = 1.93; 61–120 days, OR = 1.57). These results suggest that the incidence of gout is associated with transient increases in the incidence of cardiovascular events, and the prevention and immediate treatment of patients with gout with cardiovascular events is particularly important. However, the relationship between angina pectoris and the incidence of gout lacks detailed evidence and warrants support from high-quality clinical research, and the relationship between angina pectoris and the incidence of gout needs to be elucidated as soon as possible.

Clinical observational studies are susceptible to the risk of deviations due to statistical or environmental factors and do not substantiate the association between angina pectoris and gout flares. To further investigate the causal relationship between angina pectoris and gout, the strength of the association, and the direction of the causal relationship, a Mendelian randomization (MR) method proposed by Professor Katan [12] in 1986 has been used in this study to consider genetic variants as tool variables for testing the causal relationship between exposure factors and outcome factors. The MR method is commonly used in data analysis to assess aetiological inferences in epidemiological studies. According to Mendel's second law, this genetic variation cannot be altered by other factors; this property effectively reduces the impact of confounding factors and reverse causality and compensates for the shortcomings of clinical observational studies. The publication of a large number of pooled data related to exposure to and genetic variation between diseases has made it feasible to estimate the cause-and-effect relationships between the investigated diseases in large samples of MR data [13]. The results of MR analysis can provide a reference for clinical practice, suggesting that clinicians need to consider the correlation between the incidence of angina pectoris and gout attacks in patients, do a good job of preventing these diseases in advance, and optimise patient treatment and management processes.

## 2. Materials and Methods

### 2.1. Overall Study Design

**Figure 1:**
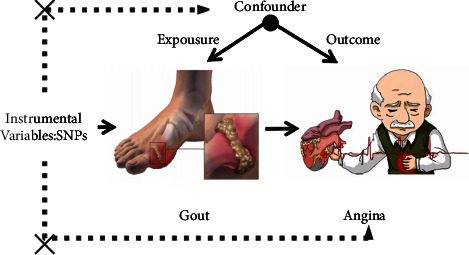
Genetic association between gout and angina pectoris; schematic diagram of a bidirectional Mendelian randomization (MR) design.

A two-sample MR method was used to analyse the causal relationship between gout and angina pectoris, with gout as the exposure factor and angina pectoris as the outcome variable. Considering that gout is characterized by an increase in blood uric acid levels, this study chose blood uric acid levels as an auxiliary exposure factor. Single nucleotide polymorphisms (SNPs) were designated as instrumental variables (IVs). Using SNPs as a modelling approach, similar to that used in randomised controlled trials, can help identify causal associations between exposure traits, such as gout, and outcome traits, especially angina. Specifically, based on publicly available human genome information from genome-wide association studies (GWAS), information on SNPs with statistically significant value related to gout and blood uric acid levels was screened as a genetic instrumental variable; information on angina was subsequently obtained through another survey derived from the Finnish database (FinnGen R9), which identified the presence of relevant SNPs; finally, using the screened SNPs, multiple MR methods were used to infer the association between gout and risk of angina (Figure 1).

### 2.2. Data Sources

Gout-related (ukb-a-107) genetic data were derived through pooled data from independent European populations from the Neil Labs IEU open GWAS database (https://gwas.mrcieu.ac.uk/; 4807 ncas and 332352 ncols). Genetic data on blood uric acid levels (bbj-a-57) is sourced from BioBank Japan (https://gwas.mrcieu.ac.uk/datasets/bbj-a-57/). The angina (finn-b-I9_ANGINA) data used in this study were obtained from the Finnish database (The FinnGen Biobank) (https://r5.finngen.fi/pheno/I9_ANGINA; 18168 ncas and 187840 ncols) (Table 1). The data used in this study were derived from published studies that were approved by an institutional review board, and all of the participants in the original study [14] provided informed consent. Therefore, no further sanctions were required for the present study [15].

### 2.3. Basic Assumptions for Two-Sample MR

The two-sample MR method used in this study used the following three key assumptions [16]:

1. Hypothesis I: Genetic variants are significantly associated with exposure; i.e., a significant association exists between instrumental variables and gout.
2. Hypothesis II: Genetic variation is not associated with any confounder associated with exposure-outcome; i.e., instrumental variables are not associated with all confounders associated with gout and angina.
3. Assumption III: Genetic variation affects outcomes only through association with the exposure factor; instrumental variables affect outcomes only through association with gout.

### 2.4. SNP Screening of Instrumental Variables

R software was used to obtain data from websites and perform tool variable SNP screening operations. SNPs were selected based on comprehensive criteria [17–19]. First, SNPs exhibiting a robust association with gout at a genome-wide significance threshold of 5 × 10*e*−8 were included. Second, SNPs were chosen to be independent of each other, ensuring that the impact of linkage disequilibrium (LD) on the results was minimised. Specifically, a stringent criterion of *r*^2^ = 0.01, with a window size of 10000 kb, was applied to avoid bias induced by LD. Moreover, the correlation between the instrumental variables (IVs) and exposure factors was assessed using the F-statistic of the SNPs. Generally, IVs with an F-statistic >10 are considered unbiased indicators.

### 2.5. Statistical Analyses

Three different MR tests, each based on different horizontal multi-effect models, including inverse variance weighting (IVW), weighted median estimator (WME), and MR-Egger regression methods, were used. The consistency between the different methods achieved upon comparing the results obtained by these three methods improves the credibility of the research results. The “Two sample MR” (version 0.5.6) software package was applied for MR and sensitivity analysis in R (version 4.2.1). Mendelian Randomization v0.5.6: updates to an R package for performing MR analyses using summarised data.

#### 2.5.1. IVW Method

The IVW method is a classic analytical model for MR with fixed effects [20, 21]. This method provides the most accurate estimates, and the regression does not require consideration of the intercept term, using the inverse of the variance of each IV as weights to perform the fit. The weighted average of the IV effect values was considered the final result, similar to the MR maximum likelihood estimation with the lowest variance of the effect estimates. If an SNP did not conform to the IV hypothesis, a random defect IVW was used to produce a bias that measures each rate against its standard error when considering possible heterogeneity. On the premise that all IVs were valid and no pleiotropy existed, the 20 SNPs in this study were measured one by one using the ratio method as well as the weighted regression method to obtain the overall estimate.

#### 2.5.2. Weighted Median Method

To satisfy the premise of effective instrumental variables, the WME method requires an effective IV of >50%. After sorting the included SNPs according to their weights, the median is considered the result. The causal estimates obtained by this method provide good agreement [22].

#### 2.5.3. MR-Egger Regression Method

If the genetic tool does not depend on the pleiotropic effect, an estimate of the effect can be obtained through MR-Egger regression. The MR-Egger regression method [23–25] uses the reciprocal of outcome variance as the weight for fitting; it also adds an intercept term to evaluate the pleiotropic effect simultaneously with regression. If the MR-Egger intercept is not significantly different from zero, the directional multiplication effect cannot be proved.

### 2.6. Sensitivity Analysis

The MR-Egger method, Cochran's *Q* test, and leave-one-out test were used to evaluate the sensitivity of the results. Additionally, an investigation of published GWAS data revealed no significant associations between the SNPs associated with gout and any other phenotypes, except for gout itself, indicating that the assumptions underlying the third MR analysis were upheld without violation. In terms of MR analysis, the second hypothesis assumes that SNPs exert their influence only by modifying interest exposures, without involving other confounding pathways. To evaluate the multidirectional nature of the direction of the SNPs, the intercept and corresponding *P* values were obtained using the MR-Egger regression method. When the intercept was almost zero, the result obtained was close to the IVW. Generally, if there is a large gap between the intercept term and 0, horizontal pleiotropy may exist between IVs [26]. The differences among IVs were examined using Cochran's *Q* test. The greater the differences, the stronger the heterogeneity. Funnel plots can effectively illustrate the directional horizontal pleiotropy of IVs by assigning a single Wald ratio for each SNP. The leave-one-out method eliminates SNPs one by one and calculates the effect generated by the combination of other units to clarify the effect of a single SNP on the outcome and evaluates the degree and stability of the impact. The results are expressed as odds ratio (OR) and 95% confidence intervals (95% CI). *P* < 0.05 was considered statistically significant [27].

### 2.7. Meta-Analysis

To comprehensively evaluate the relationship between gout and angina, we considered using blood uric acid levels as an auxiliary exposure tool. We used the “Meta” software package to perform meta-analysis on the IVW results of ukb-a-107 (gout) and bbj-a-57 (blood uric acid levels). Perform heterogeneity test analysis on the obtained results, and when *I*^2^ ≤ 50%, it indicates no significant heterogeneity; when *I*^2^ > 50%, it indicates significant heterogeneity, and heterogeneity source analysis should be conducted; and *P* < 0.05 is the standard for statistically significant differences.

## 3. Results

### 3.1. Status of IVs Related to Gout and Blood Uric Acid Levels

Following the exclusion of LD, a total of 17 IVs associated with gout were acquired, and the SNPs were characterized according to the information provided (Table 2). Similarly, we obtained 32 instrumental variables related to blood uric acid levels (Table 3). POS and CHR represent the gene location and chromosomal information, respectively; EAF signifies the frequency of effect alleles; EA/OA represents the allele; *β* signifies the effect size of gout-related SNPs; SE represents the standard error of the *β*-value; *F*-value is a statistical measure employed to evaluate the impact of weak IVs; and *P* value signifies the level of association between the SNPs and gout. All genetic tools related to gout and blood uric acid levels had a genome-wide significance level (*P* < 5 × 10*e*−8; *F* > 10), indicating that the results were not biased by weak IVs, suggesting the reliability of the present results.

### 3.2. Effects of the Results of the MR Analysis of Gout and Blood Uric Acid Levels on Angina Pectoris

**Figure 2:**
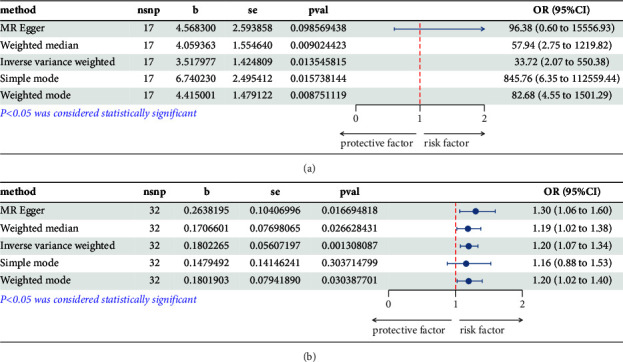
Forest plot of two-sample MR analysis of the relationship between gout/blood uric acid levels and angina pectoris.

**Figure 3:**
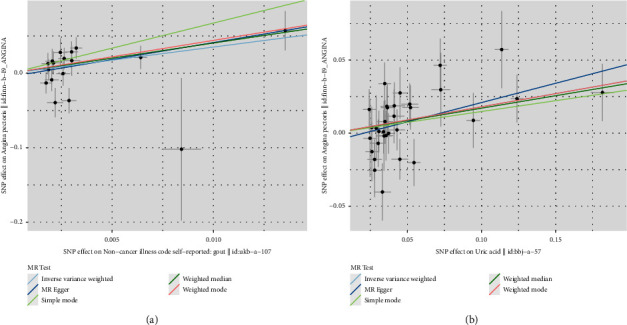
Scatter plot of the two-sample MR analysis results of the relationship between gout/blood uric acid levels and angina pectoris.

The effects of the results of the MR analysis of gout on angina were analysed using the MR-Egger, WM, IVW, SM, and WME methods in the two-sample MR package; the results are shown in the forest plot (Figure 2(a)). The MR-Egger method (*β* = 4.568300; se = 2.593858; *P*=0.098569438) showed that gout tends to cause angina (OR = 96.38; 95% CI = 0.6–15556.93), although not statistically significant. The IVW method (*β* = 3.517977; SE = 1.424809; *P*=0.013545815) showed that gout results in an increased risk of angina pectoris (OR = 33.72; 95% CI = 2.07–550.38). The results of the WME method showed that gout is significantly associated with the development of angina (*β* = 4.059363; SE = 1.554640; *P*=0.009024423; OR = 57.94; 95% CI = 2.75–1219.82). The results of the MR-Egger, WM, IVW, SM, and WME analysis of blood uric acid levels were shown in the forest plot (Figure 2(b)). The MR-Egger method (*β* = 0.2638195; Se = 0.10406996; *P*=0.016694818), indicating that high blood uric acid levels have a significant tendency to cause angina (OR = 1.30; 95% CI: 1.06–1.60). The IVW method (*β* = 0.1802265; Se = 0.05607197; *P*=0.001308087), high blood uric acid levels can lead to an increased risk of angina (OR = 1.20; 95% CI: 1.07–1.34). The WME showed a significant correlation between high blood uric acid levels and the incidence of angina pectoris (*β* = 0.1706601; Se = 0.07698065; *P*=0.026628431; OR = 1.19; 95% CI: 1.02–1.38). A scatter plot was used to observe the consistency among the results obtained from each assessment method, and the causal association among the estimates from the IVW, MR-Egger, and WME methods was similar, as judged by the slopes of the straight lines. Moreover, the IVW results prevailed, indicating that the MR results were accurate. People with gout have an increased risk of angina pectoris, and there exists a causal relationship between the two (Figure 3(a)). Similarly, the scatter plot of blood uric acid levels shows similar causal estimates for IVW, MR Egger, and weighted median methods, indicating that the MR results are accurate. High blood uric acid levels increase the risk of angina, and there is a correlation between the two (Figure 3(b)).

### 3.3. Sensitivity Analysis

#### 3.3.1. Gene Pleiotropy Tests

The intercept of the MR-Egger method regression was used to verify the presence of pleiotropy in the study. The relationship between gout and angina pectoris was tested, and the results showed that the value of the Egger intercept was −0.0045, which is close to 0, SE = 0.0091, *P*=0.6313, and MRPRESSO Global test *P*=0.11, indicating that the MR results do not face interference by the gene pleiotropy effect. Similarly, the test results for the relationship between blood uric acid levels and angina pectoris showed that the Egger interval value was −0.0055, which is close to 0, SE = 0.0057, *P*=0.3477, MRPRESSO Global test *P*=0.351, indicating that there is no interference from multiple gene effects in the MR results.

#### 3.3.2. Heterogeneity Test

**Figure 4:**
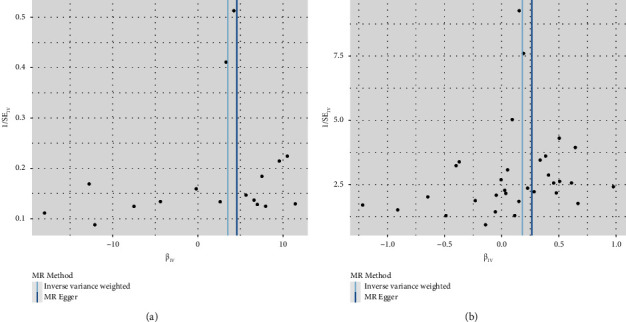
Funnel plot of the results of the heterogeneity test for MR method analysis of the relationship between gout/blood uric acid levels and angina pectoris.

Cochran's *Q* test of the relationship between gout and angina pectoris showed that the Q value and *Q* p of IVW and MR-Egger regression were 25.2367 (0.0658) and 24.8393 (0.0521), respectively, and the *P* value was >0.05, indicating that there was no heterogeneity among the SNPs. The distribution of causal effects shown by each SNP in the funnel plot was symmetrical (Figure 4(a)). Therefore, the results obtained from the 17 SNPs as IVs were relatively stable and not subject to bias by potential factors. Similarly, the Cochran Q-test for the relationship between blood uric acid levels and angina showed that the Q-values and Qp of IVW and MR-Egger regression were 34.6053 (0.2997) and 33.5863 (0.2977), respectively. *P* values greater than 0.05 indicate that there is no heterogeneity in SNPs. The distribution of causal effects shown in the funnel plot for each SNP is basically symmetrical (Figure 4(b)). Therefore, the results obtained by using 32 SNPs as instrumental variables are relatively stable and not subject to bias caused by potential factors.

#### 3.3.3. “Leave-One-Out” Rejection Tests

**Figure 5:**
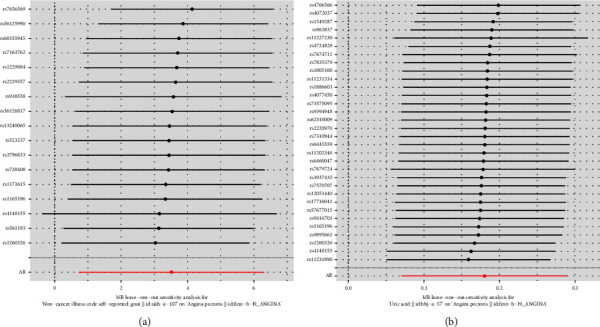
The forest map based on the results of the “leave-one-out” test of the relationship between gout/blood uric acid levels and angina pectoris.

In the MR analysis of the relationship between gout and angina pectoris, sensitivity analysis was performed using the leave-one-out method. After removing one SNP at a time, the remaining 16 SNPs were used as genetic IVs and subsequently analysed using IVW effect analysis to determine whether individual SNPs would affect the analysis results. After removing individual SNPs sequentially, the IVW effect values of the remaining SNPs did not fluctuate considerably, and all of them were close to the position of the red dots in the figure, with all *P* > 0.05, indicating that there was no SNP with strong influence on the results in the IVs. Moreover, there were no SNPs in the variables that had a strong effect on the results, indicating that the results obtained by the previous IVW method were stable and reliable (Figure 5(a)). Similarly, in the MR analysis of the relationship between blood uric acid levels and angina pectoris, the leave-one-out method showed that after sequentially removing individual SNPs, the IVW effect values of the remaining 31 SNPs did not show significant fluctuations, all close to the red dots in the figure, and the *P* value results were all greater than 0.05, indicating that the results obtained by the IVW method were stable and reliable (Figure 5(b)).

### 3.4. Meta-Analysis

**Figure 6:**
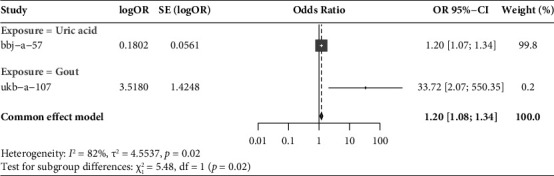
Forest plot of meta-analysis of IVW results on the relationship between gout/blood uric acid levels and angina pectoris.

Gout is a crystal associated joint disease caused by the deposition of monosodium urate, directly related to hyperuricemia caused by purine metabolism disorders and/or decreased uric acid excretion. In order to comprehensively evaluate the relationship between gout and angina, while using gout as the primary exposure tool, we considered using blood uric acid levels as an auxiliary exposure tool. In this study, we conducted a meta-analysis on the IVW results of ukb-a-107 (gout) and bbj-a-57 (blood uric acid levels) (OR = 1.20; 95% CI: 1.07–1.34, *P*=0.02), indicating that gout characterized by high blood uric acid levels significantly increases the risk of angina (Figure 6). Heterogeneity testing showed significant heterogeneity among the studies (*P*=0.02 < 0.05, *I*^2^ = 82%). The bbj-a-57 reflects blood uric acid levels; From the perspective of effect size, the bbj-a-57 effect is relatively small; The overall sample size of bbj-a-57 is relatively small, and the sample comes from East Asia, which together leads to the existence of heterogeneity. However, in this study, it can be preliminarily determined that both gout and blood uric acid levels are risk factors for angina, which further confirms that gout is a risk factor for angina and high blood uric acid levels play a mediating role between the two.

## 4. Discussion

The IVW, WME, and MR-Egger regression methods were used to infer the causal relationship between angina pectoris and the onset of gout using two-sample MR methods for the included SNPs and to test the accuracy and stability of the obtained results. Moreover, the IVW analysis of variance (AVW), WME, and MR-Egger regression methods were used for the inclusion of the included SNPs, and the accuracy and stability of the results were examined. The IVW method [OR = 33.72 (95% CI: 2.07–550.38, *P*=0.0135 < 0.05)]. The WME method (OR = 57.94 (95% CI = 2.71–1236.38, *P*=0.0093)) results showed that people suffering from gout had an increased risk of the prevalence of an angina attack. Moreover, the scatter plot showed that the causal association estimates of the IVW, MR-Egger, and WME methods were similar and that the IVW results prevailed, indicating that the MR results were accurate. Horizontal pleiotropy was assessed using the MR-Egger regression method, which indicated that there was no horizontal pleiotropy (egger intercept = −0.004466, *P*=0.631). IVW (*P*=0.066) and MR-Egger regression (*P*=0.052), as demonstrated by the Cochran Q-test, suggested the absence of heterogeneity. The results of the leave-one-out sensitivity analysis showed that after eliminating each SNP turn by turn, the IVW analysis results of the remaining SNPs were similar to those of the inclusion of all SNPs; moreover, no strongly influential SNP sites were found, i.e., the SNPs would not have a nonspecific effect on the results. Through the funnel plot, we observed that the scatter of causal association effects was symmetrically distributed, indicating no potential bias in the results of MR analysis and that the results were reliable and stable. Considering that gout is characterized by an increase in blood uric acid levels, this study chose blood uric acid levels as an auxiliary exposure factor. The results of IVW method (OR = 1.20; (95% CI: 1.07–1.34, *P*=0.001308087 < 0.05)) and WME method (OR = 1.19; 95% CI: 1.02–1.38, *P*=0.026628431 < 0.05) indicate that high blood uric acid levels increase the risk of angina attacks. At the same time, the scatter plot shows that the causal association estimates of IVW method, MR-Egger method, and weighted median method are similar, and the IVW result is accurate, indicating that the MR result is accurate. The MR-Egger regression method was used to evaluate horizontal pleiotropy, and it was found that there was no horizontal pleiotropy (egger interval = −0.0045, *P*=0.631 > 0.05). According to Cochran *Q* test, IVW (*P*=0.2997 > 0.05) and MR-Egger (*P*=0.2977 > 0.05) indicate no heterogeneity. The sensitivity analysis results of the leave-one-out method showed that after sequentially removing each SNP, the IVW analysis results of the remaining SNPs were similar to the analysis results of including all SNPs, and no strongly influential SNP sites were found, that is, SNPs did not have a nonspecific impact on the results. On the funnel plot, we can observe that the scatter plot of causal correlation effects is basically symmetrically distributed, indicating that there is no potential bias in the MR analysis results, and the results are reliable and stable. Further, we conducted a meta-analysis on the IVW results of the gout (ukb-a-107) and blood uric acid levels (bbj-a-57) (OR = 1.20; 95% CI: 1.07–1.34, *P*=0.02), indicating that gout characterized by high blood uric acid levels significantly increases the risk of angina.

Previous observational studies have shown that cardiovascular events such as angina pectoris are closely related to the occurrence of gout [3, 4], and an increasing number of patients have unstable angina combined with gout [28]. A prospective study has suggested that the two may share a similar inflammatory process. hs-CRP and IL-6-mediated inflammatory response are common pathological processes in both, and IL-6 not only accelerates the formation of unstable plaques in collaboration with angiotensin II but also participates in the acute phase of gouty attacks [29, 30]. In addition to the disease itself, related therapeutic agents, including gout and cardiovascular therapeutic agents, have an interactive effect on both diseases. Gout is a metabolic disease that is closely associated with various diseases, especially cardiovascular disease. Therefore, anti-inflammatory therapy may play a role in controlling and stabilizing the clinical course of both diseases. The anti-inflammatory effects of colchicine in cardiovascular disease have received increasing attention in recent years [31]. Higher doses of colchicine may have stronger anti-inflammatory effects on cardiovascular diseases and better clinical efficacy; however, the role and safety of different doses of colchicine in patients with unstable angina combined with acute gout need to be further explored.

The specific mechanism through which gout increases angina attacks has not been elucidated by scientific studies to date. Both diseases, angina and gout, exhibit complex pathogenetic mechanisms that are related to immune and genetic factors and may interact with each other. In addition to traditional cardiovascular risk factors, inflammation is an important risk factor for cardiovascular disease. Gout is a common inflammatory disease, and gouty attacks are characterized by acute neutrophil-predominant inflammation caused by the activation of NLRP3 inflammasome [32]. Blockade of the NALP3 inflammasome was found to prevent the recurrence of cardiovascular events in a randomised clinical trial [33]. Therefore, clarifying the causal relationship between angina and gout development and assessing the possibility of promoting the development of both could help in the precise prevention and control of angina combined with gout. In addition, gout is a disease caused by purine metabolism disorders and/or reduced uric acid excretion. Its clinical characteristics include elevated blood uric acid, recurrent episodes of characteristic acute arthritis, formation of gouty stones, chronic gouty stone arthritis, and possible involvement of the kidneys. By adopting a reasonable low purine diet and appropriate medication treatment, lower blood uric acid levels at 360 *μ*mol/L, it is an important goal to eliminate joint swelling and pain, reduce the frequency of gout attacks, and protect internal organs [34, 35].This study further considered and confirmed that high blood uric acid levels significantly increase the risk of angina attacks. Some scholars have shown that uric acid is an important independent marker of coronary artery spasm angina [36]. Clinical data analysis of 124 patients diagnosed with stable or unstable angina and chronic complete occlusion by coronary angiography revealed a correlation between high uric acid levels and poor collateral circulation in the coronary arteries [37]. The virtual histology intravascular ultrasound was used to evaluate the noncoronary atherosclerotic lesions of 119 patients with angina pectoris during percutaneous coronary intervention and 8 months after statin treatment; Statin therapy alleviates coronary atherosclerosis and reduces blood uric acid levels [38]. Karabağ et al. confirmed that serum uric acid levels are associated with SYNTAX score II and long-term mortality in patients with multi vessel lesions and/or unprotected left main trunk lesions receiving percutaneous coronary intervention [39]. Yang et al. found a positive correlation between blood uric acid and the Gensini score of coronary artery stenosis in patients with coronary heart disease, which is an independent predictive factor for evaluating the degree of coronary artery stenosis [40].

## 5. Conclusion

This study used a two-sample MR method to infer the causal relationship between angina pectoris and gout attacks and preliminarily concluded that a positive causal relationship exists between the two. Considering that gout is characterized by an increase in blood uric acid levels, this study also confirmed that high blood uric acid levels increase the risk of angina attacks. Although the specific mechanisms have not been elucidated, this study enhances the thinking on the risk factors for the onset of these two diseases. Although the specific mechanism was not elucidated, the findings enhance the consideration of the risk factors for the development of these two diseases. Patients with gout often experience cardiovascular diseases in combination, and few guidelines have recommended drug treatment programs. We expect that subsequent studies will focus on the group of patients with gout combined with angina pectoris and report on the risk factors for angina pectoris in patients with acute gout, as well as the anti-inflammatory and analgesic treatment programs for the two diseases, to reduce the acute attacks of angina pectoris in patients with gout and provide a basis for decision-making in terms of clinical drug treatment. In addition, strengthening the early screening for the onset of angina pectoris in patients with gout as well as the timely prevention and treatment of the disease is of significance in controlling the prevalence of the disease. However, our research still has limitations. First, our MR analysis found that gout increases the risk of angina, and uric acid, as a mediating factor, is particularly important in the study of the mechanism between gout and angina. Further analysis revealed that blood uric acid increases the risk of angina. However, there is some heterogeneity in the results of meta-analysis, and further confirmation with more datasets and samples should be considered in subsequent studies to reduce heterogeneity. Second, the incidence of gout and angina is higher in males than females, but our data are all from public databases and cannot be analysed for subgroups of specific factors such as age and gender. Thirdly, all subjects in the GWAS data are of European or East Asian ancestry, and further research is needed to determine whether the results can be extended to other populations. Fourthly, increasing the sample size of GWAS can enhance the intensity of IV. We need larger scale GWAS for in-depth research and support from clinical multicenter epidemiological studies.

## Figures and Tables

**Table 1:** Summary information of genome-wide association study (GWAS) data in Mendelian randomization research.

Exposure or outcome	GWAS ID number	Ethnic origin	Sample size	Year	Number of SNPs
Angina pectoris	finn-b-I9_ANGINA	European	206008	2021	16380426
Gout	ukb-a-107	European	337159	2017	10894596
Blood uric acid	Bbj-a-57	East Asian	109029	2019	6108953

**Table 2:** Status of gout-related instrumental variables.

SNP	Position	chr	EA	OA	eaf.exposure	beta.exposure	se.exposure	*P* value exposure	*F*
rs1165196	25813150	6	A	G	0.567643	0.00263457	0.000289627	9.38*E − *20	82.74492997
rs1171615	61469090	10	T	C	0.768861	0.00244908	0.000340697	6.57*E* − 13	51.67367205
rs1229984	100239319	4	C	T	0.977549	−0.0084261	0.000969401	3.57*E* − 18	75.55205667
rs1260326	27730940	2	C	T	0.607251	−0.00322976	0.000293591	3.83*E* − 28	121.0194087
rs13240065	73015369	7	A	G	0.12925	−0.00301105	0.000427462	1.87*E* − 12	49.61818885
rs2229357	57843711	12	A	G	0.244676	−0.00258249	0.000333565	9.81*E* − 15	59.93994592
rs36126817	122528460	12	G	A	0.478406	−0.00187091	0.000287229	7.35*E* − 11	42.4276737
rs3796833	10012878	4	T	C	0.278855	0.00210912	0.000319964	4.35*E* − 11	43.45105694
rs4148155	89054667	4	G	A	0.113515	0.0135423	0.000451317	1.48*E* − 197	900.3709527
rs523237	64330324	11	C	A	0.691455	−0.00183543	0.000311327	3.74*E* − 09	34.75698356
rs561193	64353029	11	G	A	0.285423	0.00300301	0.000318097	3.73*E* − 21	89.12394671
rs56125990	69742387	16	G	A	0.154068	0.00219353	0.000398525	3.71*E* − 08	30.29535291
rs68155945	89270328	4	A	G	0.45344	−0.00174476	0.000289851	1.75*E* − 09	36.2344594
rs7163762	90658679	15	A	G	0.229467	0.00202371	0.000343308	3.76*E* − 09	34.74791159
rs738408	44324730	22	T	C	0.216187	−0.00204893	0.00034818	3.99*E* − 09	34.62953028
rs7656569	89166761	4	A	C	0.193483	−0.00287406	0.000367476	5.25*E* − 15	61.16932667
rs938558	9939205	4	A	G	0.726799	0.00641057	0.00032167	2.56*E* − 88	397.1661029

**Table 3:** Status of instrumental variables related to blood uric acid levels.

SNP	Position	chr	EA	OA	eaf.exposure	beta.exposure	se.exposure	*P* value exposure	*F*
rs11202346	88908912	10	T	G	0.2224	0.03489	0.00528	3.89*E − *11	43.66506327
rs11227230	65356674	11	A	G	0.0778	−0.1816	0.007896	4.92*E − *117	528.9533952
rs11231334	62844587	11	C	T	0.2757	−0.04296	0.005456	3.46*E − *15	61.99830583
rs11231890	64611543	11	T	C	0.5324	−0.07209	0.004412	5.14*E − *60	266.9801935
rs1165196	25813150	6	A	G	0.8407	0.05158	0.0057	1.43*E − *19	81.88662358
rs12051440	80163600	16	T	C	0.9069	−0.04116	0.007224	1.21*E − *08	32.46349378
rs1260326	27730940	2	C	T	0.4409	−0.03472	0.00424	2.65*E − *16	67.05446778
rs1549287	69590366	16	G	A	0.8452	−0.03315	0.005792	1.05*E − *08	32.75744037
rs17716041	88832384	4	T	G	0.8241	−0.05209	0.005529	4.48*E − *21	88.75964059
rs1805100	76476396	8	A	G	0.432	0.03729	0.004247	1.63*E − *18	77.09394058
rs1886603	119482303	10	A	G	0.3744	0.02607	0.004341	1.91*E − *09	36.06637473
rs2220970	9857749	11	A	G	0.3419	0.02475	0.004434	2.39*E − *08	31.15724354
rs3937435	113406190	12	G	A	0.3543	−0.03648	0.00439	9.61*E − *17	69.05269275
rs4072037	155162067	1	T	C	0.8289	−0.04491	0.00559	9.45*E − *16	64.54498353
rs4077450	79931595	16	T	G	0.5862	−0.03561	0.004707	3.86*E − *14	57.2341913
rs4148155	89054667	4	G	A	0.2955	0.1136	0.00464	2.00*E − *132	599.4054697
rs4724828	1298448	7	T	C	0.4123	0.02793	0.005015	2.57*E − *08	31.01701476
rs4766566	111706877	12	T	C	0.6775	0.05443	0.00446	2.97*E − *34	148.9384916
rs57677015	10422356	4	T	G	0.1267	0.0725	0.006325	2.05*E − *30	131.3877736
rs62310009	89250957	4	C	A	0.1979	0.03427	0.005284	8.88*E − *11	42.06322731
rs6445559	53099466	3	G	A	0.4376	−0.02594	0.004399	3.67*E − *09	34.77218804
rs6460047	73042443	7	C	T	0.106	−0.04096	0.006978	4.38*E − *09	34.45545312
rs7310944	113045997	12	A	G	0.6318	−0.029	0.004393	4.11*E − *11	43.57863163
rs73575095	79750332	16	C	T	0.2808	−0.03388	0.004709	6.26*E − *13	51.76418998
rs7570707	61451744	2	T	C	0.3729	0.02428	0.00437	2.74*E − *08	30.86984799
rs7674711	9916180	4	C	T	0.7052	−0.09447	0.004599	9.41*E − *94	421.9500198
rs7679724	9985376	4	T	G	0.586	0.1239	0.004284	5.56*E − *184	836.4571319
rs7835379	95975080	8	A	G	0.7546	0.0303	0.004928	7.78*E − *10	37.80453136
rs9394948	43334755	6	C	A	0.6594	−0.03079	0.004536	1.13*E − *11	46.0758426
rs9416703	60283008	10	C	A	0.4751	0.03615	0.004289	3.53*E − *17	71.04025485
rs963837	30749090	11	C	T	0.3432	−0.02785	0.004867	1.05*E − *08	32.74369653
rs9895661	59456589	17	T	C	0.4776	0.04506	0.004821	9.03*E − *21	87.35909209

## Data Availability

All data in this study can be obtained through the corresponding author's e-mail, E-mail: acuresearch@126.com.
